# Detection and clearance of a mosquito densovirus contaminant from laboratory stocks of Zika virus

**DOI:** 10.1590/0074-02760180432

**Published:** 2019-02-07

**Authors:** Allan Henrique Depieri Cataneo, Diogo Kuczera, Ana Luiza Pamplona Mosimann, Emanuele Guimarães Silva, Álvaro Gil Araújo Ferreira, João Trindade Marques, Pryscilla Fanini Wowk, Claudia Nunes Duarte dos Santos, Juliano Bordignon

**Affiliations:** 1 Fundação Oswaldo Cruz-Fiocruz, Instituto Carlos Chagas, Laboratório de Virologia Molecular, Curitiba, PR, Brasil; 2 Universidade Federal de Minas Gerais, Instituto de Ciências Biológicas, Departamento de Bioquímica e Imunologia, Belo Horizonte, MG, Brasil

**Keywords:** arbovirus isolation, contamination, C6/36 cultures, densovirus, vertebrate cells, Zika virus

## Abstract

**BACKGROUND:**

The Zika virus (ZIKV) epidemics that affected South America in 2016 raised several research questions and prompted an increase in studies in the field. The transient and low viraemia observed in the course of ZIKV infection is a challenge for viral isolation from patient serum, which leads to many laboratories around the world sharing viral strains for their studies. C6/36 cells derived from *Aedes albopictus* larvae are commonly used for arbovirus isolation from clinical samples and for the preparation of viral stocks.

**OBJECTIVES:**

Here, we report the contamination of two widely used ZIKV strains by *Brevidensovirus*, here designated as mosquito densovirus (MDV).

**METHODS:**

Molecular and immunological techniques were used to analyse the MDV contamination of ZIKV stocks. Also, virus passages in mammalian cell line and infecting susceptible mice were used to MDV clearance from ZIKV stocks.

**FINDINGS:**

MDV contamination was confirmed by molecular and immunological techniques and likely originated from C6/36 cultures commonly used to grow viral stocks. We applied two protocols that successfully eliminated MDV contamination from ZIKV stocks, and these protocols can be widely applied in the field. As MDV does not infect vertebrate cells, we performed serial passages of contaminated stocks using a mammalian cell line and infecting susceptible mice prior to re-isolating ZIKV from the animals’ blood serum. MDV elimination was confirmed with immunostaining, polymerase chain reaction (PCR), and analysis of the mosquitoes that were allowed to feed on the infected mice.

**MAIN CONCLUSIONS:**

Since the putative impact of viral contaminants in ZIKV strains generally used for research purposes is unknown, researchers working in the field must be aware of potential contaminants and test viral stocks to certify sample purity.

In the past few years, human Zika virus (ZIKV) infection has caused an increase in public health concerns due to an association with new clinical manifestations, such as *Guillain-Barré* syndrome and congenital neurological manifestations.^1^ ^,^ ^2^ These concerns accelerated scientific research aimed at understanding the mechanisms by which the ZIKV interacts with its host to cause new clinical presentations.

Between 1947, when ZIKV was first reported in a Uganda forest, and 2015,^3^ 124 articles were published regarding ZIKV. However, recent outbreaks and clinical manifestations associated with ZIKV infection resulted in more than 4,500 Zika-related published medical/scientific manuscripts during the 2016/2018 period. This increase in research was beneficial to the ZIKV field and added to our understanding of this new, emerging viral disease.

Arboviral isolation from clinical samples typically employs the use of mosquito cells, such as C6/36, from *Aedes albopictus* larvae.^4^ It is well known that mosquito cell lines can harbor contaminants including insect viruses, and the presence of contaminant viruses could induce cytopathic effects in insect cells, including syncytia formation or cell lysis, depending on the contaminant virus.^5^ Viruses belonging to the genus *Brevidensovirus* are among previously reported insect cell culture contaminants.^6^ ^,^ ^7^ *Brevidensovirus* is a genus of the *Parvoviridae* family, *Densovirinae* sub-family, which encompasses viruses known for infecting insects of the *Diptera* order, like *Aedes aegypti* and *Ae. albopictus*, among others.^8^ As far as it is known, these viruses are not able to replicate in vertebrates, however they can be pathogenic for their invertebrate hosts.^9^ ^,^ ^10^ ^,^ ^11^

In this study, we identified the presence of two different *Brevidensovirus*, here designated as mosquito densovirus (MDV), contaminating two ZIKV strains; one strain is of African origin, and the other strain is of Asian lineage. These strains were sent to our laboratory for research purposes. We also provide two simple strategies to remove MDV contamination from ZIKV strains using vertebrate cells as a bottleneck for MDV replication.

## MATERIALS AND METHODS

*Virus stocks production* - Two different ZIKV strains were recently sent to our laboratory. The ZIKV strain of Asian origin was named *strain A*, and the strain that resembled African origin was *strain B*. As those samples were sent to our laboratory without information on viral passage history we identified both samples of ZIKV *strains A* and *strain B* as zero (P.0). Both P.0 viral supernatants were used to infect C6/36 cells (ATCC^®^ CRL-1660™) that were cultured in L-15 media supplemented with 5% FBS, 25 µg/mL gentamicin and 0,26% triptose (Thermo Fisher Scientific, Grand Island, New York, USA) at a multiplicity of infection (MOI) of 0.01 for viral stock production.

*ZIKV titration using foci forming assay* - ZIKV virus titers were determined by the foci forming immunodetection assay in C6/36 cells (FFU_C6/36_/mL), as previously described.^12^ Briefly, C6/36 cells were infected with 10-fold serially diluted mice sera / cell culture supernatant for 90 minutes. After inoculum was removed a CMC overlay media (L-15 plus 5% SFB, 0.26% tryptose, 25 µg/mL gentamicin, 1.6% carboxymethylcellulose) was added and plates incubated at 28ºC for seven days. The immunostaining was performed using the anti-flavivirus mouse monoclonal antibody 4G2 (anti-E protein; ATCC^®^ HB-112™), followed by alkaline phosphatase conjugated goat anti-mouse antibody (Promega, Madison, WI, USA). The reaction was detected using NBT/BCIP substrate solution (nitroblue tetrazolium chloride/5-bromo-4-chloro-39-indolyphosphate p-toluidine salt) (Promega, Madison, WI, USA). Foci were counted and expressed as FFU_C6/36_/mL.

*Molecular detection of densovirus* - Briefly, viral nucleic acids from C6/36 cell supernatants infected with each ZIKV strain were isolated using the RNeasy Mini kit (QIAGEN). For MDV DNA amplification (324 bp), the primers DNV3R (5’-TTTATTTCCATAGATATTGACTGTTTCGAT-3’) and DNV3F 5’-AATCGAGAAACAGCATACTACACATTCGT-3’) were used as previously described.^13^ These primers amplified a viral genomic region encompassing a small segment of the NS1 and NS2 genes of MDV. As a control for MDV amplification, a plasmid containing the same target gene from the MDV BR/07 isolate was used.

Additionally, a reverse transcription polymerase chain reaction (RT-PCR) assay was used for the molecular detection of MDV. Briefly, total nucleic acids from the supernatant and pellet of C6/36 cells was extracted using TRIzol reagent (Invitrogen). Blood samples from ZIKV infected mice were collected one to four days post inoculation, and nucleic acids was extracted using TRIzol reagent (Invitrogen). A total of 500 ng of nucleic acids was reverse transcribed using 300 ng of random primers. The resulting cDNA was used as a template for PCR with the primers DensoBR07_F (5’-ATTGTTGGGAGCATGACGGA-3’) and DensoBR07_R (5’-CAACGGTTTGACCAGCGAAA-3’) resulting in 212 bp of amplification. To test for the presence of densovirus in the mosquitoes that fed on ZIKV infected mice, the total nucleic acids from individual mosquitoes was extracted and pooled to prepare cDNA. During the replication cycle of MDV the ssDNA genome produces mRNA,^14^ thus, both RT-PCR or direct PCR could be used to detect MDV contamination (data not shown).

*Zika virus detection by RT-PCR* - ZIKV genomic RNA was detected by RT-PCR (364 bp) using the primer set ZIKVENVF (5’-GCTGGDGCRGACACHGGRACT-3’) and ZIKVENVR (5’-RTCYACYGCCATYTGGRCTG-3’) as previously described.^15^ ^,^ ^16^ RNA from the ZIKV strain ZV BR2015/15261 isolate (South Brazil, 2016) was used as a control for ZIKV E gene amplification.

*Immunofluorescence assay for ZIKV and MDV detection* - C6/36 cells (2x10^4^ cells/well) were seeded in a 96-well plate and infected (in triplicate) with P.0 of ZIKV *strain A* and *strain B* at an MOI of 1. The MOI was based on the titration of ZIKV *strain A* and *strain B* in C6/36 using a pan-flavivirus monoclonal antibody that recognises the E protein (4G2; ATCC^®^ HB-112™; see ZIKV titration using foci forming assay). After 72 h, the cells were fixed and permeabilised with methanol:acetone (v/v) as previously described.^13^ For immunostaining, three different antibodies were used - an anti-flavivirus envelope (E) protein (4G2), an *in-house* mouse polyclonal antibody anti-MDV and an anti-MDV monoclonal antibody (clone 94DL1; IgG2a kappa).^13^ A goat anti-mouse IgG Alexa Fluor 488 conjugate was used as secondary antibody, and digital images were taken with a fluorescence microscope (Leica DMI6000B) using LAS AF (Leica) software. As an MDV positive control, C6/36 cells were infected with MDV BR/07 (GenBank: GU452720) with a multiplicity of genome (MOG) of 0.01 or 1 for 72 h. The polyclonal and monoclonal antibodies against MDV strain BR/07 used in the immunostaining assays were generated in a previous study.^13^

*Densovirus nucleic acid sequencing* - PCR fragments (324 bp) from MDV detection were purified using the High Pure PCR Product Purification Kit (Roche), and nucleotide sequencing was performed with primers for DNV3R and DNV3F by dideoxynucleotide termination sequencing at Macrogen Inc. (Seoul, South Korea). The sequences were assembled using the Assembler tool (http://www.hpabioinformatics.org.uk/cgibin/assembly_tool/seq_assemble.cgi?no=2) and aligned using ClustalW^17^ as implemented in BioEdit software v.7.2.5.^18^ The length of nucleotide sequence used in the analysis was 265 bp due to the primer sequence removal. The consensus sequence of densovirus *strain A* and *B* were deposited in GenBank under accession numbers, MH720336 and MH720337, respectively.

*MDV removal from ZIKV samples* - As MDV does not infect vertebrate cells, we performed serial passages of ZIKV *strain A* P.0 and *strain B* P.0 in A549 cells (lung epithelial cells; ATCC: CCL185). Briefly, A549 cells (1x10^5^ cells/well in 24 well plates) were infected with 100 µL of ZIKV *strain A* P.0 or *strain B* P.0 for 90 min. After infection, cell monolayers were washed three times with 1X PBS and incubated in culture medium (DMEM-F12, 7% FCS, 100 IU/µg/mL of penicillin/streptomycin) for 72 h. The cell culture supernatants were collected and used (100 µL) to infect a new set of A549 cell cultures (second passage). An additional passage in A549 cells was performed as previously described (third passage). To confirm the exclusion of MDV after three passages in A549 cells, the cell culture supernatant of ZIKV *strain A* P.3/A549 and *strain B* P.3/A549 was passaged three additional times in the C6/36 mosquito cell line. All A549 and C6/36 cell passages were performed as described above. Nucleic acid was extracted from cell supernatants, and RT-PCR and PCR were performed for ZIKV and MDV, respectively.

Additionally, ZIKV *strain A* was used to infect A129 mice using a dose of 4x10^6^ PFU^19^ per individual by the intraperitoneal route. Blood samples were collected daily from 1 to 4 days post infection (dpi), and the presence of MDV was tested as previously described. To certify that MDV was eliminated in mouse blood, 3 dpi *Ae. aegypti* females (5-7-day-old) were allowed to feed on ZIKV infected animals. MDV RT-PCR was performed on mosquitoes at four days post feeding. A total number of 10 fed mosquitoes were used to test for the presence of MDV. Additionally, ZIKV was titrated in mice sera (using foci forming assay in C6/36 cells) three days after infection in order to quantify ZIKV recovery.

**Fig. 1:**
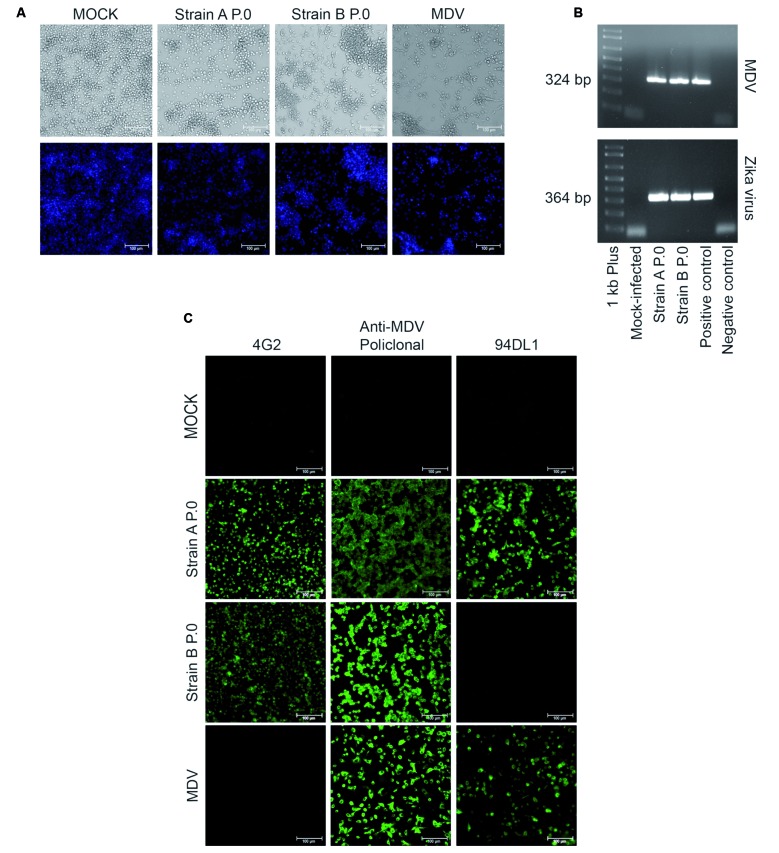
mosquito densovirus (MDV) coinfection in Zika virus (ZIKV) strains A P.0 and B P.0. (A) Cytopathic effects in C6/36 MDV-contaminated ZIKV strain A and strain B cells compared to mock-infected cells and C6/36 cells infected with MDV BR/07 at an multiplicity of genome (MOG) of 1 for 72 h. (B) Agarose gel showing polymerase chain reaction (PCR) amplification of a 324 bp fragment from the MDV genome and reverse transcription-PCR (RT-PCR) amplification of a 364 bp fragment of the ZIKV E gene in strain A and strain B (P.0). (C) Immunofluorescence assay of ZIKV strain A and strain B (P.0) infected C6/36 cells stained with a 4G2 monoclonal antibody, anti-MDV mouse polyclonal serum, and monoclonal antibody (clone 94DL1) raised after immunisation with the MDV BR/07 strain. As a positive control, C6/36 cells were infected with MDV BR/07 at an MOG of 1 for 72 h.

*ZIKV infection to confirm MDV elimination* - C6/36 cells were infected with a low [0.01] to high [10] MOI of ZIKV *strain A* P.3/C6/36 and ZIKV *strain B* P.3/C6/36. After 72 h, the supernatants and cell pellets were tested for the presence of MDV using a PCR assay as previously described.

*Ethics* - Experiments involving A129 mice were approved by the ethics committee at UFMG (CEUA 337/2016).

## RESULTS

Two different ZIKV strains (referred to as *strain A* and *strain B*) were recently sent to our laboratory from two different sources for research purposes. During the preparation of viral stocks, visual inspection of C6/36 cell cultures revealed an atypical cytopathic phenotype that raised suspicion of potential contamination with an additional virus/microorganism (Fig. 1A). Our previous experience with MDV contamination^13^ showed similar cytopathic effects, so we performed molecular and immunological assays to check for possible contamination.

Using PCR, we amplified a segment of the MDV genome in ZIKV *strains A* and *B* to confirm coinfection with MDV (Fig. 1B); an immunofluorescence assay was also performed. The immunofluorescence assay confirmed the presence of the ZIKV E-antigen in the cytoplasm and coinfection of both ZIKV *strains A* and *B* with MDV (Fig. 1C). Furthermore, the inability of an anti-MDV monoclonal antibody to recognise *strain B* P.0 suggests that different MDV strains were coinfecting the ZIKV strains. To address this, we determined the nucleotide sequence (from PCR product) of the MDV present in both ZIKV strains. Despite the short viral genomic region analysed (265 bp), the nucleotide identity was 95.4% between the MDVs present in each ZIKV strain, which confirmed different viral strains. This could be explained by 1) the different passage history of the two ZIKV strains; 2) divergent evolution of both MDV strains due to the maintenance in C6/36 cell culture. However, we could not confirm any of these hypotheses once we do not have information on the ZIKV *strain A* and *B* passage history before samples were sent to our Laboratory. Despite that, results suggest that the contamination originated from two different sources (Table).

A comparison of the new MDV isolates with the MDV previously reported by our group (BR/07; GenBank: GU452720) shows a nucleotide identity of 98.4% with the sequence amplified from *strain A* and an identity of 96.2% with the one amplified from *strain B* P.0 (Table). It is important to note that the C6/36 cell cultures in our laboratory are routinely checked for insect viral contaminations, including MDV, due to our reference laboratories activities for the Brazilian Ministry of Health.

**Fig. 2:**
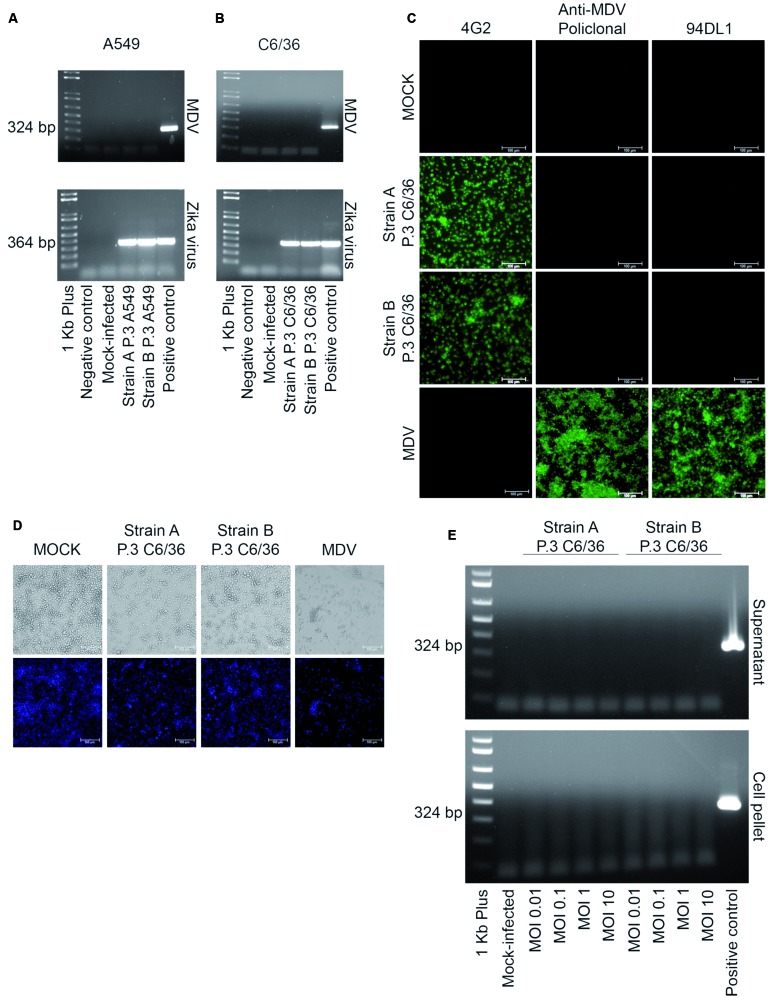
mosquito densovirus (MDV) clearance from the Zika virus (ZIKV) strains A and B (P.0). Agarose gels showing polymerase chain reaction (PCR) amplification of a 324 bp fragment from MDV and reverse transcription-PCR (RT-PCR) amplification of a 364 bp ZIKV E gene fragment in ZIKV strain A and strain B after three passages in A549 cells (P.3/A549) (A) followed by three passages in C6/36 cells (P.3/C6/36) (B). (C) Immunofluorescence assay in C6/36 cells infected with ZIKV strain A and strain B (P.3/C6/36) after three passages in C6/36 cells stained with 4G2 monoclonal antibody, anti-MDV mouse polyclonal serum, and anti-MDV monoclonal antibody (clone 94DL1). (D) Cytopathic effects on C6/36 cells infected with ZIKV strain A and strain B after three passages in C6/36 compared to mock-infected cells. Infection with the MDV BR/07 strain multiplicity of genome (MOG) 0.01 for 72 h was used as positive control for immunofluorescence and cytopathic effect assays. (E) Agarose gel showing PCR amplification of a 324 bp fragment from MDV. C6/36 cells were infected with different multiplicity of infections (MOIs) (0.01, 0.1, 1 and 10) of ZIKV strain A and strain B after three passages in C6/36 cells (P.3 C6/36). Nucleic acid was extracted from cell pellet and cell culture supernatant of infected cells and tested for the amplification of MDV genes by PCR. As a positive control, a plasmid containing the same target gene from the isolate MDV BR/07 was used (324 bp).

MDV belongs to the *Parvoviridae* family and the *Brevidensovirus* genus.^14^ This nonenveloped virus presents a 4kB negative-polarity, single-stranded DNA genome.^20^ MDV is considered nonpathogenic for humans; however, MDV may be detrimental to mosquitoes.^21^ ^,^ ^22^ Once we confirmed contamination of each ZIKV strain with MDV, we focused on strategies to eliminate it from the ZIKV samples to prevent interference with future experiments. It was previously demonstrated that MDV does not infect vertebrate cells,^6^ so we performed serial passages of the ZIKV *strains A* and *B* using a ZIKV-susceptible A549 lung epithelial cell line.^23^ After three passages of ZIKV *strains A* and *B* in A549 cells, the MDV coinfection was no longer detected in cell culture supernatants using PCR, while detection of a ZIKV envelope gene was successful (Fig. 2A). Additionally, PCR for MDV and RT-PCR for ZIKV were performed after each passage (P1, P2 and P3) using nucleic acids extracted from the supernatants, and the results demonstrated that fragments of the NS1 and NS2 genes of MDV were not detected for *strain A* after the first passage (P1) in A549 cells or at the second passage (P2) for *strain B* (data not shown).

Additionally, to confirm the exclusion of MDV from ZIKV strains, we performed three additional passages of ZIKV *strain A* P.3/A549 and *strain B* P.3/A549 using the C6/36 mosquito cell line, as this cell line is susceptible and permissive to MDV. After the third passage in C6/36 cells, nucleic acid was extracted from the supernatants, and RT-PCR and PCR for ZIKV and MDV, respectively, were performed (Fig. 2B). These results demonstrated that successive passages of MDV-contaminated ZIKV strains in A549 cells are effective for removing MDV contamination from ZIKV samples (Fig. 2). An immunofluorescence assay was also used to confirm MDV exclusion from each ZIKV-strain (Fig. 2C). Additionally, ZIKV titration after each passage in cell culture showed a ZIKV recovery rate between 10^4^ to 10^7^ FFU/mL (data not shown). After passages in A549 and C6/36 cells, the cytopathic effects observed in C6/36 cells were no longer apparent compared to previous infections prior to the removal of MDV (Figs 1A and 2D). As infection with ZIKV induces cytopathic effects on C6/36 even after the elimination of MDV, some damage on C6/36 cells could be observed when compared to mock-infected cells (Fig. 2D).

To further confirm the elimination of MDV from each ZIKV strain, C6/36 cells were infected with a different MOI of ZIKV *strain A* P.3/C6/36 and ZIKV *strain B* P.3/C6/36. After 72 h, the supernatants and cell pellets were tested for MDV using PCR. Even after infection with a high MOI [10], MDV was no longer detected in these ZIKV stocks. These results confirmed the efficiency of this protocol in the removal of MDV contamination from ZIKV strains (Fig. 2E).

**TABLE:** Nucleotide identity matrix comparing the two mosquito densovirus (MDV) presented on Zika virus (ZIKV)-isolates

	GU452720	ZIKV *strain A*	FJ805445	ZIKV *strain B*
GU452720	100%	98.4%	97.3%	96.2%
ZIKV *strain A*	98.4%	100%	96.6%	95.4%
FJ805445	97.3%	96.6%	100%	98.8%
ZIKV *strain B*	96.2%	95.4%	98.8%	100%

GU452720: mosquito densovirus BR/07; FJ805445: Culex densovirus 0507JS11.

**Fig. 3:**
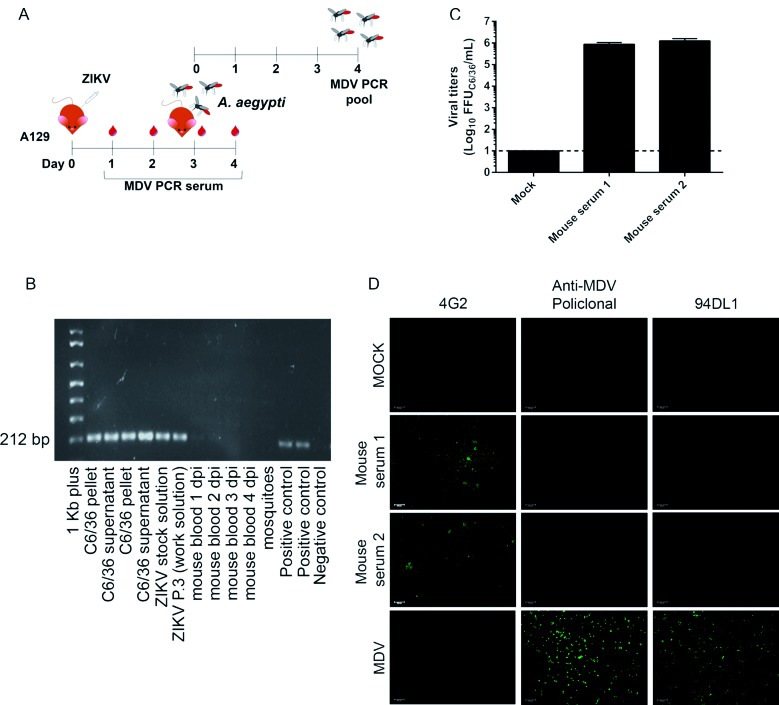
infection of A129 mice provide a reliable strategy for clearance of mosquito densovirus (MDV)-contamination. (A) Experimental design for in vivo assays. (B) Agarose gel showing reverse transcription-PCR (RT-PCR) amplification of a 212 bp fragment from MDV in the blood of A129 mice infected with 4x106 PFU of Zika virus (ZIKV) strain A at different days post infection (dpi) and in Aedes aegypti mosquitoes that fed on infected animals. As a control, ZIKV strain A stocks and C6/36 cells (pellets and culture supernatants were used) together with a plasmid control were tested for MDV. (C) Viral titers in mice sera three days post-infection. The sera from two mice were tested in biological replica. (D) Immunofluorescence assay of C6/36 cells infected with ZIKV strain A recovered from mice sera three days post-infection. C6/36 cells were infected with mice sera at a multiplicity of infection (MOI) of 1 and after three days stained with 4G2 monoclonal antibody, anti-MDV mouse polyclonal serum, and anti-MDV monoclonal antibody (clone 94DL1). As a positive control, C6/36 cells were infected with MDV BR/07 at a multiplicity of genome (MOG) of 0.01 for 72 h.

As an alternative protocol, we also hypothesised that passing ZIKV isolates in susceptible mice would eliminate MDV. To test this hypothesis, type I IFN receptor KO (A129) mice were infected with contaminated stocks of ZIKV *strain A* (Fig. 3A)*.* Blood was collected from one to four dpi and tested for MDV. As early as 1 dpi and throughout the kinetics, blood samples were negative for MDV. We also allowed *Ae. aegypti* to feed on the blood of infected mice at 3 dpi to further test for the successful elimination of MDV (since it would be amplified in mosquitoes even if present at low titers). Mosquitoes that fed on infected mice were negative for MDV (Fig. 3B). These results indicate that the passage of MDV-contaminated ZIKV-stocks in mice is also a suitable method to eliminate contamination. For all time points, the presence of MDV was tested in mice and mosquitoes, and we confirmed ZIKV RNA using RT-PCR (data not shown). Additionally, ZIKV was recovered from mice sera at higher titers three days after infection (Fig. 3C). Also, C6/36 cells were infected with mice sera (three dpi) and an immunofluorescence assay confirmed the elimination of MDV contamination and the recovery of ZIKV *strain A* (Fig. 3D).

## DISCUSSION

The exchange of biological samples, such as viral isolates and cell lines, often occurs between research laboratories. Although this is important for scientific development, the certification of the microorganism strains or cell lines shared between laboratories is essential to avoid contamination problems. One of the most notorious cases of biological contamination in research laboratories is the contamination of cell lines with HeLa cells.^24^ Contamination of cell lines with MDV is not unusual, as this has already been demonstrated in the mosquito cell lines C6/36 and AP-61.^7^ Although difficult to track, we suspect that the MDV contamination origin began in contaminated cell cultures used for virus propagation prior to both ZIKV strains being exhaustively shared between laboratories.

Although MDV infection can result in the development of cytopathic effects, the virus can also be unnoticed due to its ability to establish persistent infections without any clear cytopathic effects.^6^ ^,^ ^7^ Multiple authors have also demonstrated the ability of MDV to affect cell growth that is likely due to arrest of the cell cycle at the G2 phase.^13^ ^,^ ^25^ Thus, it has been suggested that MDV could be used to control the mosquito population and have implications for the transmission of arboviruses.^11^ ^,^ ^22^

There are no studies addressing the effects of coinfections with MDV and ZIKV, and the outcome of such a coinfection is unknown; however, the negative impact of MDV in dengue virus infection and replication was demonstrated *in vitro* and *in vivo*, further reinforcing the potential use of MDV for the biological control of arboviral infections.^11^ ^,^ ^13^ Given the similarities between DENV and ZIKV, it is plausible that MDV and ZIKV coinfection may affect *in vitro* and *in vivo* ZIKV infections. Conversely, studies using the C6/36 cell line and *Ae. aegypti* mosquito models have shown that coinfections with the chikungunya and densonucleosis viruses do not impact the infection and replication of either virus.^26^ Furthermore, MDV could induce the production of antibodies in BALB/C mice after immunisation with Freund’s complete adjuvant (first dose) and Alu-S-Gel (doses 2 to 4).^13^ Thus, the potential impact of infecting mice with flaviviruses (dengue or ZIKV) contaminated with MDV is still an open question.

Regarding the protocol used for MDV clearance from ZIKV stocks, additional care should be considered. First, it has been already shown that vertebrate cells and some supplies used in cell culture, like fetal calf serum, could also harbor contaminant viruses.^27^ ^,^ ^28^ A contamination with Infectious Bovine Rhinotracheitis Virus (IBRV) has already been shown in a commercially A549 cell seed stock.^28^. Ideally, the source of cells used for virus growth and titration should be tested for the presence of contaminants. Also, for virus evolution studies, the protocols suggested here could impact on the results, as they are based in successive viral passaging in cell culture or mice, which could introduce genetic mutations in ZIKV genome. It has been shown that a single passage in cells could influence the genetic diversity of Chikungunya virus.^29^ Furthermore, successive passages of ZIKV in vertebrate (Vero cells) or invertebrate (C6/36 cells) cells influence plaque sizes, kinetic and restriction to grow. In addition, four mutations were identified associated with plaque size that might have influence on ZIKV biology.^30^

Since coinfections are not limited to ZIKV strains, researchers who work in the arbovirology field should check their cell lines and viral stocks periodically to avoid contamination with arthropod viruses such as MDV. The main purpose of reporting these findings is to call the attention of the scientific community of the potential presence of mosquito virus contaminants in ZIKV strains/stocks. We also suggest two simple strategies to efficiently eliminate MDV contamination from ZIKV strains/stocks, *in vitro* and *in vivo* passages in vertebrate cell lines or mice models, respectively. Finally, the potential interference of MDV contamination in ZIKV isolates needs further analysis.
